# Neutrophil-lymphocyte ratio predicts pathologic tumor response and survival after preoperative chemoradiation for rectal cancer

**DOI:** 10.1186/1471-2482-14-94

**Published:** 2014-11-18

**Authors:** Ik Yong Kim, Sei Hwan You, Young Wan Kim

**Affiliations:** Department of Surgery, Yonsei University Wonju College of Medicine, 162 Ilsan-dong, Wonju-si, Gangwon-do (220-701) Korea; Department of Radiation Oncology, Yonsei University Wonju College of Medicine, 162 Ilsan-dong, Wonju-si, Gangwon-do (220-701) Korea

**Keywords:** Neutrophil-lymphocyte ratio, Preoperative chemoradiation, Rectal neoplasm

## Abstract

**Background:**

Neutrophil-lymphocyte ratio (NLR) reflects the balance between pro- and anti-tumor immune activities. We evaluated whether NLR is associated with pathologic tumor response and prognosis in rectal cancer patients that underwent preoperative chemoradiaton therapy (CRT) with surgery.

**Methods:**

One hundred two patients with rectal cancer that were treated by preoperative CRT followed by surgery were enrolled. A total of 50.4 GY of radiation and 5-FU-based chemotherapy were delivered. An NLR ≥ 3 was considered to be elevated. Pathologic tumor response based on ypTNM stage was categorized into two groups, good response (n = 35, pathologic complete response and ypTNM I) and poor response groups (n = 67, ypTNM II, III, and IV).

**Results:**

Twenty-five patients (24.5%) had elevated NLR. Multivariate analysis showed that an elevated CEA level (p = 0.001), larger tumor (p = 0.03), and elevated NLR (p = 0.04) were significant predictors for a poor response. Poor pathological tumor response and elevated NLR were risk factors for cancer-specific and recurrence-free survivals.

**Conclusion:**

An elevated NLR before CRT can be used as predictors for poor tumor response and unfavorable prognostic factors. Dominant pro-tumor activities of neutrophils or reduced anti-tumor immune response by lymphocytes, as determined by NLR, may have a impact on poor tumor response and unfavorable prognosis.

**Electronic supplementary material:**

The online version of this article (doi:10.1186/1471-2482-14-94) contains supplementary material, which is available to authorized users.

## Background

Preoperative chemoradiation (CRT) followed by total mesorectal excision (TME) is advocated as a treatment for locally advanced rectal cancer [1]. However, it is important to identify patients based on their responsiveness to preoperative CRT. Patients with pathologic complete responses would have favorable oncologic outcomes, and identification of patients with poor response could prevent unnecessary radiation and surgery delay [2, 3]. Diverse clinical and molecular predictors for preoperative CRT have been investigated, but there is currently no clear consensus about reliable markers for pathologic tumor response after preoperative CRT.

Inflammation is closely associated with tumorigenesis. Colorectal cancers are infiltrated by various immune cells such as neutrophils, T and B lymphocytes, dendritic cells, macrophages, natural killer cells, and mast cells [4]. These cells produce cytokines, chemokines, and inflammatory mediators. Tumor-host interactions cause a systemic inflammatory response, which leads to changes in levels of circulating white blood cells (WBC) [5]. In recent years, high neutrophil-lymphocyte ratio (NLR), increased levels of neutrophils and decreased levels of lymphocyte have been suggested to be poor prognostic factors in colorectal cancer [6, 7].

To date, there have been few studies examining the role of NLR as a predictor for pathologic tumor response and as a prognostic factor after preoperative CRT in rectal cancer. It has been suggested that anti-tumor immune response is part of the tumor cell death process induced by ionizing radiation [8]. Accordingly, tumor shrinkage may be caused by the host immune response and can be a direct effect of radiation. Thus, we postulated that host immune status as determined by NLR could be used as a predictor for tumor response after preoperative CRT in rectal cancer. The aim of this study was to evaluate the correlation of pretreatment NLR with pathologic tumor response and prognosis in rectal cancer patients who have undergone preoperative CRT with TME.

## Methods

### Patients

From October 1996 to November 2012, 111 patients with rectal cancer who were treated with preoperative CRT followed by TME were enrolled in this observational study. Patients with histologically confirmed rectal adenocarcinoma within 15 cm from the anal verge were included. Endorectal ultrasonography, computed tomography scan and pelvic magnetic resonance imaging were used for preoperative staging. All patients had stage T3 or T4 and/or node-positive disease and underwent a TME-based major surgery after CRT. Nine patients were excluded because they did not complete scheduled chemotherapy or radiation therapy and did not undergo radical resection. Finally, a total of 102 patients were analyzed in this study. Informed consent was obtained from all patients, and this study was approved by the Institutional Review Board (No. YWNR-13-5-059).

Patient follow-up lasted until death or until the cut-off date of February 28, 2013. Ten patients were lost to follow-up. The median follow-up interval was 39 months (range, 3–238 months).

The primary objective of this study was to evaluate the correlation of pretreatment NLR with pathologic tumor response and prognosis. Pathologic tumor response based on ypTNM stage was categorized into two groups as follows; good response group (n = 35, pathologic complete response and ypTNM I) versus poor response group (n = 67, ypTNM II III, and IV). Two patients were diagnosed as stage IV after CRT and were included in this study. The STROBE guidelines were used to ensure the reporting of this study (Additional file 1) [9].

### Systemic inflammatory markers

Blood samples were obtained within 7 days before CRT. WBC, neutrophil, lymphocyte, platelet counts, C-reactive protein (CRP) and albumin levels were recorded. NLR was calculated as the neutrophil count divided by the lymphocyte count using preoperative blood test results. Post-CRT NLR data were obtained from laboratory results approximately four weeks after CRT. An NLR ≥3 was considered elevated. The modified Glasgow prognostic score (mGPS) was recorded as follows: score 0, CRP ≤10 mg/l; score 1, CRP >10 mg/l and albumin ≥35 g/l; score 2, CRP >10 mg/l and albumin <35 g/l [10].

### Preoperative chemoradiation

#### Preoperative CRT

All patients underwent three-dimensional conformal treatment planning using computed tomography scan simulation. The total radiation dose was 50.4 Gy. Radiation was delivered to the whole pelvis (45 Gy in 25 fractions) with a boost to the primary tumor (5.4 Gy in 3 fractions) over 5 weeks [11]. Intravenous chemotherapy (425 mg/m^2^ 5-fluorouracil and 20 mg/m^2^ leucovorin) was administered during weeks 1 and 5 of radiation therapy.

### Surgery, pathology, and adjuvant therapy

TME was performed by a single surgeon four to eight weeks after CRT was completed [12]. A standardized pathologic examination was performed, and the TNM staging system, ypT and ypN were recorded according to the American Joint Committee on Cancer (AJCC).

Three to eight weeks after the surgery, adjuvant chemotherapy (400–425 mg/m^2^ intravenous 5-fluorouracil and 20 mg/m^2^ leucovorin) was performed for five days, every 28 days for four cycles.

### Statistical analysis

Statistical analysis was performed using IBM SPSS Statistics for Windows, Version 20.0 (IBM, Armonk, NY, USA). Both student’s t-test for continuous variables and Chi-square test (Fisher's exact test) for categorical variables were performed. Logistic regression was used to identify the pathologic tumor response predictors. Survival analysis was performed using the Kaplan-Meier method with the log rank test and Cox proportional hazard model. A P-value less than 0.05 was considered to be statistically significant.

## Results

Twenty-five patients (24.5%) had elevated NLR (≥3). The pathologic tumor response based on the ypTNM stage was categorized into two groups as follows: good response (pathologic complete response (n = 9) and ypTNM I (n = 26)) and poor response (ypTNM II (n = 34), III (n = 31), and IV (n = 2)) groups.

### Clinicopathologic characteristics

There were no differences in age, gender, tumor location, operation type, or duration from CRT to definitive surgery between the good and poor response groups. A larger tumor diameter (≥3 cm) (p = 0.003), and elevated carcinoembryonic antigen (CEA) level (≥5 ng/mL) (p = 0.003) were more common in the poor response group (Table 1).

**Table 1:** **Clinicopathologic characteristics** (**n** = **102**)

		Pathologic response ^*^		
		Good	Poor	
Variable		N (%)	N (%)	P
Age (years)	<60	17 (48.6)	36 (53.7)	0.6
	≥60	18 (51.4)	31 (46.3)	
Gender	Male	28 (80)	54 (80.6)	0.9
	Female	7 (20)	13 (19.4)	
Tumor location	Upper (10.1-15 cm)	0 (0)	3 (4.5)	0.2
	Mid (5.1-10 cm)	12 (34.3)	31 (46.3)	
	Low (<5 cm)	23 (65.7)	33 (49.3)	
Operation type	Low anterior resection	30 (85.7)	56 (83.6)	0.8
	APE or Hartmann procedure	5 (14.3)	11 (16.4)	
Duration (preoperative CRT to surgery) (week)	<6	22 (62.9)	42 (62.7)	0.99
	≥6	13 (37.1)	25 (37.3)	
Tumor diameter (cm)	<3	30 (85.7)	38 (56.7)	0.003
	≥3	5 (14.3)	29 (43.3)	
ypT classification	0	9 (25.7)	4 (6.0)	<0.001
	1	5 (14.3)	1 (1.5)	
	2	21 (60.0)	4 (6.0)	
	3	0 (0)	55 (82.1)	
	4	0 (0)	3 (4.5)	
ypN classification	0	35 (100)	36 (53.7)	<0.001
	1	0 (0)	22 (32.8)	
	2	0 (0)	9 (13.4)	
CEA (ng/mL)	<5	29 (85.3)	35 (55.6)	0.003
	≥5	5 (14.7)	28 (44.4)	

*Good response group: pathologic complete response and ypTNM I, poor response group: ypTNM II, III and IV.

APE, abdominoperineal excision; CRT, chemoradiation; CEA, carcinoembryonic antigen.

### Hematological characteristics

The mean white blood cell (p = 0.04) and neutrophil counts (p = 0.01) were higher in the poor response group. There were no differences in the mean lymphocyte or platelet counts. An elevated pre-CRT NLR (≥3) was identified more frequently in the poor response group (p = 0.001), and the mean level of CRP was higher in the poor response group (p = 0.03). An elevated post-CRT NLR (≥3) and mGPS of 1 or 2 was more common in the poor response group; however, this difference was not statistically significant (p = 0.4 and p = 0.2, respectively) (Table 2).

**Table 2:** **Hematological characteristics**

		Pathologic response ^*^			
		Good	Poor		
		Mean ± SD	Mean ± SD	P	Reference range
White blood cell		7 ± 2	7.9 ± 2	0.04	(4.0–10.0) × 10^9^ /L
Neutrophil		4.1 ± 1.5	4.9 ± 1.7	0.01	(1.8–7.5) × 10^9^ /L
Lymphocyte		2.1 ± 0.7	2.1 ± 0.7	0.96	(1.0–2.8) × 10^9^ /L
Pre-CRT NLR	<3	33 (94.3%)	44 (65.7%)	0.001	
	≥3	2 (5.7%)	23 (34.3%)		
Post-CRT NLR	<3	20 (57.1%)	31 (47.7%)	0.4	
	≥3	15 (42.9%)	34 (52.3%)		
Platelet		275 ± 42	315 ± 113	0.2	(165–360) × 10^9^ /L
C-reactive protein		0.4 ± 0.3	1.4 ± 2.7	0.03	<0.03 mg/dL
Albumin		4.2 ± 0.4	4 ± 0.5	0.2	(3.3-6.1) g/dL
mGPS	0	33 (94.3%)	58 (86.6%)	0.2	
	1,2	2 (5.7%)	9 (13.4%)		

*Good response group: complete pathologic response and ypTNM I, poor response group: ypTNM II,III and IV.

SD, standard deviation; CRT, chemoradiation therapy; NLR, neutrophil-lymphocyte ratio; mGPS, modified Glasgow prognostic score.

### Multivariate analysis of pathologic tumor response predictors

After extensive univariate analysis, only significant variables (CEA, tumor diameter, NLR, neutrophil count, and CRP) were included in the multivariable logistic regression models. Logistic regression analysis showed that an elevated CEA level (≥5 ng/mL) (p = 0.001), large tumor diameter (≥3 cm) (p = 0.03), and elevated NLR (≥3) (p = 0.04) were significant predictors of poor pathologic response (Table 3). Neutrophil count (p = 0.6) and CRP (p = 0.6) were not significant.

**Table 3:** **Predictors for poor pathologic tumor response** (**ypTNM II**, **III**, **and IV**): **multivariate analysis**

		HR (95% CI)	P
CEA (ng/mL)	<5	1	0.001
	≥5	13.2 (2.8 - 62.1)	
Tumor diameter (cm)	<3	1	0.03
	≥3	3.6 (1.2 - 11.8)	
NLR	<3	1	0.04
	≥3	5.2 (1.1 - 26.5)	
C-reactive protein (mg/dL)		1.7 (0.3 - 11.8)	0.6
Neutrophil count (× 10^9^ /L)		1.1 (0.8 - 1.6)	0.6

HR, hazard ratio; CI, confidence interval; CEA, carcinoembryonic antigen; NLR, neutrophil-lymphocyte ratio.

### Survival analysis

**Figure 1:**
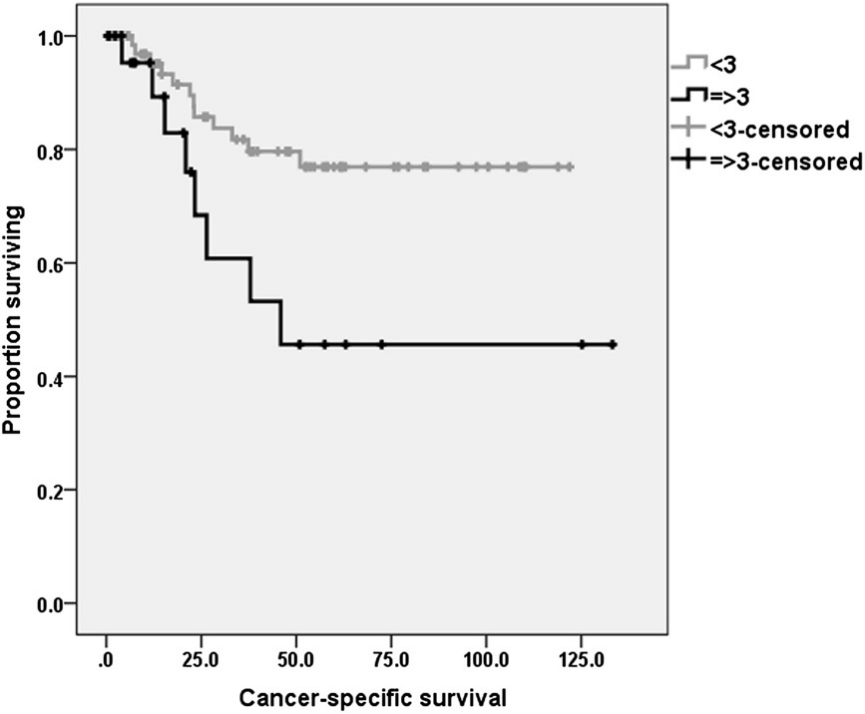
**Cancer** **-** **specific survival rates according to neutrophil** **-** **lymphocyte ratio** **(** **NLR** **).** The 5-year cancer-specific survival rates were 76.9% and 45.6% in patients with NLR < 3 and NLR ≥ 3, respectively (p = 0.01).

**Figure 2:**
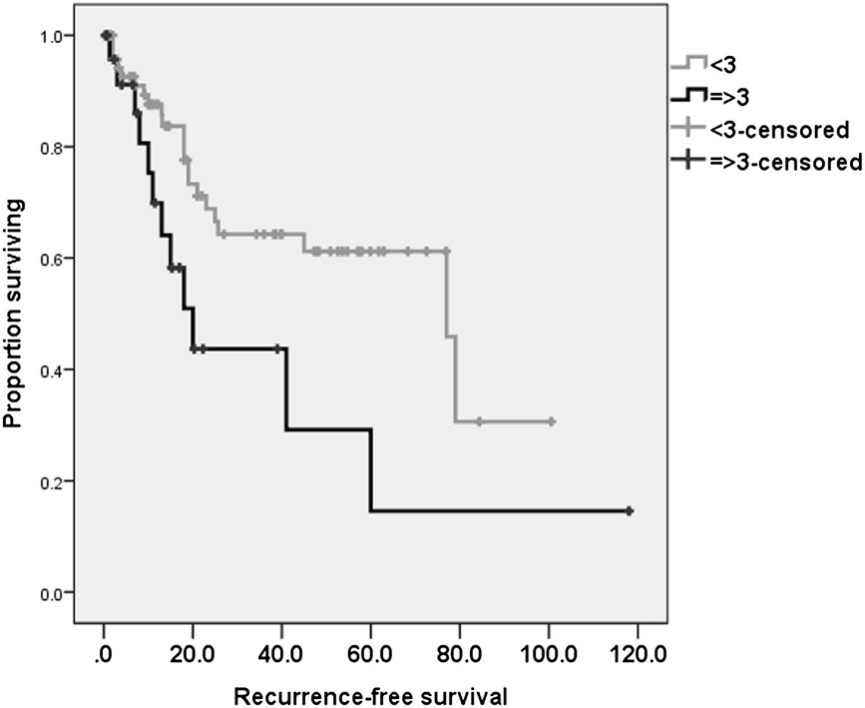
**Recurrence** **-** **free survival rates according to neutrophil** **-** **lymphocyte ratio** **(** **NLR** **).** The 5-year recurrence-free survival rate was 61.2% and 14.6% in patients with NLR < 3 and NLR ≥ 3, respectively (p = 0.01).

There were 20 cases of cancer-specific deaths and 34 cases of recurrences. In terms of cancer-specific survival, poor pathologic tumor response based on ypTNM (II, III, and IV) and elevated CEA level, and NLR were significant risk factors in univariate analysis. The 5-year cancer-specific survival rates were 76.9% and 45.6% in patients with NLR < 3 and NLR ≥ 3, respectively (p = 0.01) (Figure 1). The 5-year recurrence-free survival rate was 61.2% and 14.6% in patients with NLR < 3 and NLR ≥ 3, respectively (p = 0.01) (Figure 2).

Multivariate analysis using the Cox proportional hazard model showed that poor tumor response (hazard ratio (HR) = 10, p = 0.03) and elevated NLR (HR = 6.6, p = 0.02) were significant prognostic factors (Table 4). In terms of recurrence-free survivals poor tumor response (HR = 3.4, p = 0.001 and HR = 3.1, p = 0.002) and elevated NLR (HR = 3.6, p = 0.01 and HR = 2.8, p = 0.03) were adverse prognostic factors in both univariate and multivariate analyses (Table 5).

**Table 4:** **Risk factors associated with cancer**-**specific survival**

		Univariate		Multivariate	
		HR (95% CI)	p	HR (95% CI)	P
Age (years)	< 60 vs. ≥ 60	1.1(0.4 − 2.7)	0.8	NA	
Gender	Male vs. female	1 (0.3 − 2.9)	0.9	NA	
Tumor location	Upper, mid vs. low	2.3 (0.9 − 5.6)	0.1	NA	
Operation type	LAR vs. APE or Hartmann	1 (0.3 − 3)	1	NA	
Duration (preoperative CRT to surgery)(week)	<6 vs. ≥6	0.5 (0.2 − 1.4)	0.6	NA	
Tumor diameter (cm)	<3 vs. ≥3	1.9 (0.8 − 4.5)	0.2	NA	
ypTNM	pCR,I vs. II,III,IV	11 (1.5 − 85)	0.02	10 (1.3 − 75)	0.03
CEA (ng/mL)	< 5 vs. ≥ 5	3.3 (1.4 − 8.2)	0.01	1.8 (0.7 − 4.7)	0.2
NLR	< 3 vs. ≥ 3	10 (2–49)	0.01	6.6 (1.3 − 32)	0.02

HR, hazard ratio; CI, confidence interval; NA, not applied; LAR, low anterior resection; APE, abdominoperineal excision; CRT, Chemoradiation; pCR, pathologic complete response; CEA, carcinoembryonic antigen; NLR, neutrophil-lymphocyte ratio.

**Table 5:** **Risk factors associated with recurrence**-**free survival**

		Univariate		Multivariate	
		HR (95% CI)	p	HR (95% CI)	P
Age (years)	< 60 vs. ≥ 60	1.2 (0.6 − 2.4)	0.5	NA	
Gender	Male vs. female	1.2 (0.5 − 2.6)	0.7	NA	
Tumor location	Upper, mid vs. low	1.4 (0.7 − 2.7)	0.4	NA	
Operation type	LAR vs. APE or Hartmann	0.6 (0.3 − 1.6)	0.4	NA	
Duration (preoperative CRT to surgery) (week)	<6 vs. ≥6	0.8 (0.4 − 1.6)	0.5	NA	
Tumor diameter (cm)	<3 vs. ≥3	1.5 (0.8 − 3.1)	0.2	NA	
ypTNM	pCR,I vs. II,III,IV	3.4 (1.7 − 6.8)	0.001	3.1 (1.5 − 6.2)	0.002
CEA (ng/mL)	< 5 vs. ≥ 5	1.7 (0.8 − 3.7)	0.2	1.3 (0.6 − 2.9)	0.5
NLR	< 3 vs. ≥ 3	3.6 (1.5 − 8.9)	0.01	2.8 (1.1 − 6.8)	0.03

HR, hazard ratio; CI, confidence interval; NA, not applied; LAR, low anterior resection; APE, abdominoperineal excision; CRT, Chemoradiation; pCR, pathologic complete response; CEA, carcinoembryonic antigen; NLR, neutrophil-lymphocyte ratio.

## Discussion

The major finding of this study is that NLR prior to preoperative CRT can predict pathologic tumor response in patients with rectal cancer. In addtion, elevated NLR was a risk factor for recurrences-free and cancer-specific survivals.

Neutrophils have a pro-tumor effect on the tumor microenvironment and can influence the environment throughout the stages of tumor progression. Secreted cytokines and chemokines mediate inflammatory cell recruitment, tumor growth, angiogenesis and adaptive immune response suppression [13]. In comparison, lymphocytic infiltration, predominantly CD4+ or CD8+ T cells, in the primary tumor is recognized as an anti-tumor immune response, and a prominent infiltration is associated with improved survival in colorectal cancer [14, 15]. Weak lymphocytic infiltration of the tumor margin after liver resection is linked to poor prognosis in patients with colorectal liver metastasis [16]. Thus, NLR, the ratio of neutrophils and lymphocytes, reflects the balance between pro- and anti-tumor immune activities. Elevated NLR could reflect dominant pro-tumor activities of neutrophils or reduced anti-tumor immune response by lymphocytes. These may be the reason that patients with elevated NLR showed unfavorable pathologic tumor response and prognosis in this study.

A higher lymphocyte ratio in white blood cells has been suggested as a pathologic complete response predictor after CRT in locally advanced rectal cancer [17, 18]. However, in our study, the lymphocyte counts and ratios were not significantly different when the good and poor response groups were compared. Krauthamer et al. [19] studied predictors of tumor response to preoperative CRT in rectal cancer and determined that serum albumin (>3.5 mg/dl) and NLR (<5) were predictors for complete pathologic response in clinical stage III (n = 71) patients but not in clinical stage II patients. They explained that no association between NLR and clinical stage II disease may be due to small study samples. In our study, the albumin level was not a significant factor. In addition, other clinical parameters such as small tumor diameter and normal CEA level have been shown to predict pathologic complete response after preoperative CRT in rectal cancer [20, 21], and these were also significant predictors in our study.

In regard to recurrence and survival, an elevated NLR is associated with poor outcome, and NLR has been shown as a prognostic factor in patients with resectable or unresectable colorectal cancer [22, 23]. Specifically in rectal cancer patients, NLR showed conflicting results with respect to survival. NLR was identified as an independent prognostic factor by Liu et al. [24] but was not in a study by Chiang et al. [25] However, neither study focused on patients that underwent preoperative CRT. Carruthers et al. [7] investigated 115 patients with locally advanced rectal cancer that were undergoing preoperative CRT and determined that NLR and the presence of residual tumor (R) status were predictors for recurrence and survival, but ypTNM stage was not considered in their analysis. In a study by Krauthamer et al. [19], NLR was evaluated not as a prognostic factor, but as a predictor for tumor response. The pathologic tumor stage (ypTNM) after CRT has been suggested as a reliable prognostic factor for recurrence and survival in locally advanced rectal cancer [26]. In our study, pathologic tumor response based on ypTNM stage as well as NLR was a significant risk factor for recurrence and cancer-specific survivals.

In addition to NLR, other markers of systemic inflammatory response, such as CRP and mGPS (CRP and albumin), have been shown to be risk factors for inferior survival in colorectal cancer [6, 10]. Toiyama et al. [27] investigated correlations between levels of systemic inflammation markers such as CRP and NLR in 84 patients with locally advanced rectal cancer. They observed that elevated CRP and ypTNM III stage were adverse prognostic factors for overall survival and an elevated CRP level was an independent risk factor for disease-free survival. In our study, the mean level of CRP was higher in the poor response group, but multivariate analysis showed that the CRP level was not an independent risk factor for poor pathological response. In addition, patients with mGPS of 1 or 2 were more common in the poor response group, but this did not reach statistical significance. Recently, laboratory blood data such as thrombocytosis and lymphocyte counts have been shown to have prognostic value in rectal cancer [28, 29]. Kawai et al. [29] demonstrated that pre-CRT thrombocytosis is related to poor pathological tumor regression and poor local recurrence-free survival. In our study, the mean number of platelets was higher in the poor response group, but the difference was not statistically significant. Yasuda et al. [28] observed that hematological parameters such as hemoglobin, albumin, lymphocyte percentage, platelet counts, CRP, and fibrogen level were associated with tumor response. Low lymphocyte count was an adverse risk factor for disease-free survival in their study. We did not observe a difference in lymphocyte counts between good and poor response groups.

NLR can be obtained simply and readily in a routine blood test; however, to date, there is no consensus about an NLR cut-off value. A cut-off value of 3 or 5 for NLR as a continuous variable has been studied in rectal cancer [7, 24, 25]. In this study, we chose 3 as the cutoff based on our own preliminary data analysis in which a cutoff of 3 yielded the highest predictive ability in terms of pathological tumor response and survival analysis. Thus, it is important to identify the optimal cut-off value to accurately predict pathologic tumor response and patient prognosis after CRT.

The small sample size was a limitation of this study. A growing body of evidence suggests that NLR is closely related to cardiovascular or cerebrovascular disease [30, 31]. Another limitation of our study is that we did not evaluate potential interactions between comorbid conditions and rectal cancer. However, to the best of our knowledges, this is the first study that has simultaneously explored the impact of NLR on the pathologic tumor response and prognosis in rectal cancer patients that were undergoing preoperative CRT. Based on our results, elevated NLR may be used as a predictor for advanced disease features. Accordingly, patients with elevated pretreatment NLR may be considered for more aggressive preoperative or adjuvant treatment [32, 33].

## Conclusion

In conclusion, an elevated NLR prior to preoperative CRT can be used as a poor pathologic tumor response predictor in patients with rectal cancer. An elevated NLR can identify patients at high risk for recurrence and cancer-specific death. Dominant pro-tumor activities of neutrophils or reduced anti-tumor immune response by lymphocytes, as determined by elevated NLR, may have a impact on poor tumor response and unfavorable prognosis in terms of recurrence and survival.

## Electronic supplementary material

Additional file 1: **The STROBE guidelines were used to ensure the reporting of this observational study STROBE Statement—checklist of items that should be included in reports of observational studies.** (DOC 92 KB)

## Abbreviations

NLR: Neutrophil-lymphocyte ratio
CRT: Chemoradiaton therapy
TME: Total mesorectal excision
WBC: White blood cells
CRP: C-reactive protein
MGPS: Modified Glasgow prognostic score
AJCC: American Joint Committee on Cancer
HR: Hazard ratio
CEA: carcinoembryonic antigen.
